# Combining Weighted Contour Templates with HOGs for Human Detection Using Biased Boosting

**DOI:** 10.3390/s19061458

**Published:** 2019-03-25

**Authors:** Shih-Shinh Huang, Shih-Han Ku, Pei-Yung Hsiao

**Affiliations:** 1 Department of Computer and Communication Engineering, National Kaohsiung University of Science and Technology, Kaohsiung 80778, Taiwan; poww@nkust.edu.tw; 2 Department of Electrical Engineering, National University of Kaohsiung, Kaohsiung 80811, Taiwan; pyhsiao@nuk.edu.tw

**Keywords:** HOGs, global contour template, expectation maximization, boosting

## Abstract

This paper proposes a method to detect humans in the image that is an important issue for many applications, such as video surveillance in smart home and driving assistance systems. A kind of local feature called the histogram of oriented gradients (HOGs) has been widely used in describing the human appearance and its effectiveness has been proven in the literature. A learning framework called boosting is adopted to select a set of classifiers based on HOGs for human detection. However, in the case of a complex background or noise effect, the use of HOGs results in the problem of false detection. To alleviate this, the proposed method imposes a classifier based on weighted contour templates to the boosting framework. The way to combine the global contour templates with local HOGs is by adjusting the bias of a support vector machine (SVM) for the local classifier. The method proposed for feature combination is referred to as biased boosting. For covering the human appearance in various poses, an expectation maximization algorithm is used which is a kind of iterative algorithm is used to construct a set of representative weighted contour templates instead of manual annotation. The encoding of different weights to the contour points gives the templates more discriminative power in matching. The experiments provided exhibit the superiority of the proposed method in detection accuracy.

## 1. Introduction

Detecting humans is an important topic in many applications, such as intelligent surveillance and intelligent transportation systems (ITSs) and has received considerable attention. However, vision-based human detection is still challenging due to factors including varied illumination conditions, complex backgrounds, various types of clothes, the occlusion effect, and a broad range of human poses and views. Compared to stereo vision, a monocular solution demands less computation and eases the calibration process. Therefore, we present an approach for detecting humans based on monocular vision. Since the camera is mounted on a moving platform, the background is not static so that the background subtraction approaches widely used for identifying the regions of human candidates are inapplicable in our work. The most common method of human detection in the literature is to use the sliding window strategy which formulates the detection problem as binary classification one. This scans an image pyramid by a fixed-sized window and bounding boxes around humans are then determined from the use of non-maximum suppression process.

To mitigate the difficulty from intra-class variance, shape is a kind of effective feature in representing the human appearance and determining the existence of a human in a single window or not. A comprehensive survey of the use of the shape feature can found in [1]. In the human detection literature, the schemes of shape description or modeling can be generally classified into two categories: global and local shape descriptors. In general, the description of a human shape can be achieved by using a set of representative binary-contour templates that covers a wide range of human poses and views. The presence of a human in a window is determined by comparing the extracted contour with the constructed templates. In [2,3,4], the used templates are full-body binary contours in various viewing angles and poses. In [5], only the edge points with orientation between [+45, –45] are used for representing human contour. In general, the more templates are used for describing the human shapes, the higher the accuracy in human detection that can be achieved. However, this would significantly increase the computation complexity. The ways of reducing the computation burden at the expense of little accuracy are either to extract a smaller set of representative templates [6] or to organize the templates in a hierarchical structure [2,7]. In improving the discriminative ability of templates in matching, the strategy of assigning different matching importance to contour point is proposed by [8] and [9]. Besides, the contour magnitude [3] or orientation [10,11] are also imposed in the matching stage to improve the matching accuracy. However, the global modeling approaches using contour shape have a tendency to fail in detecting partially occluded humans and are generally less flexible in dealing with shape articulations. Motivated by this, many approaches using a local shape descriptor are proposed in the human detection literature.

In this category, a detection window is divided into thousands of patches, each of which is described by local features. The modeling of the human shape using a local patch descriptor is either through feature concatenation [12,13] or feature selection. One of the well-known local shape descriptors for human detection is the histogram of oriented gradients (HOGs) firstly proposed by Dalal and Triggs [14]. The effectiveness of HOGs for human detection has been proven and the discussion of this point can be found in [15]. In order to further improve the representative capability of HOGs, many methods have been proposed in the literature. Wang et al. [16] introduced a circular type of blocks to represent head shape which is a salient human body part. In [17], they proposed symmetry weighting function instead of Gaussian kernel in HOGs representation. Besides HOGs, other local features, such as local binary pattern (LBP) [18], edgelets [19,20], shapelets [21] or combined features [22,23,24] are well known in this category. Although the use of local shape descriptors can effectively tackle the occlusion problem, they generally result in false detection in the case of complex background and noise effect. This is because they lack in modeling shape in a global manner. To complement the benefits of both local and global shape descriptors, this work aims at presenting an approach that imposes the global contour feature to the HOGs-based boosting framework.

The proposed method mainly consists of training and detection phases. Given a training dataset consisting of positive (human) and negative (non-human) ones, the first step of the training phase is to generate a set of weighted contour templates by an expectation maximization (EM) algorithm. Then, we perform template matching to obtain a matching score for further distinguishing human from non-human through a thresholding strategy. In order to alleviate the problem generally faced by the HOGs-based approach, the classification result from the template-based classifier is imposed to the HOGs-based boosting framework by adjusting the bias of the support vector machine (SVM) hyper-plane at each boosting round. We refer to this framework that systematically integrates the global contour and local HOGs features as biased boosting. At the detection stage, the template-based classifier is firstly applied to determine the presence of a human. If the answer is yes, a set of learned positive-biased weak classifiers that have the bias in favor of positive label is taken for further voting to check the existence of a human in a scanning window. Otherwise, negative-biased weak classifiers are used for voting.

The remainder of the paper is organized as follows. In Section 2, we present a way of forming a human classifier based on a set of weighted contour templates learned from the EM algorithm. Section 3 describes how to learn a human detector using the proposed biased boosting which systematically integrates the global contour and local HOGs features. Section 4 demonstrates and discusses the experiments on three popular datasets. Finally, we conclude this paper in Section 5 with some discussion.

## 2. Template-Based Classifier

Using a binary contour template to describe the human shape is popular in the literature. To improve the discriminative ability of contour templates, we impose importance(s) on the contour point(s) instead of considering them as equally weighted in the literature. The construction of the weighted contour templates is through the use of EM [25].

### 2.1. Problem Formulation

**Figure 1:**
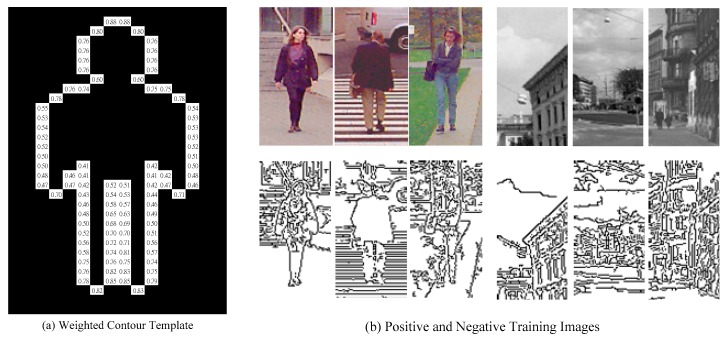
(**a**) Weighted contour template is shown in a schematic form; (**b**) some examples of positive and negative training samples are shown.

A weighted contour human template $\theta_{j}={\left\{p_{k}^{\left(j\right)},\alpha_{k}^{\left(j\right)}\right\}}_{k=1}^{\left|\theta_{j}\right|}$ is a binary contour image, where $p_{k}^{\left(j\right)}$ is the position of the *k*th contour point in $\theta_{j}$, $\alpha_{k}^{\left(j\right)}$ indicates the matching importance of $p_{k}^{\left(j\right)}$, and $\left|\theta_{j}\right|$ denotes the number of contour points in the template $\theta_{j}$. Figure 1a shows an example of a weighed template. Every white block denotes a contour point $p_{k}^{\left(j\right)}$ and the number inside the block is its associated weighting factor $\alpha_{k}^{\left(j\right)}$. The matching difference between a binary edge image *y* composed of a set of edge points and $\theta_{j}$ using Chamfer distance can be expressed as:

(1) $$d(y,\theta_{j})=\frac{1}{\left|\theta_{j}\right|}\sum_{k=1}^{\left|\theta_{j}\right|}\alpha_{k}^{\left(j\right)}DT_{y}(p_{k}^{\left(j\right)})$$

$DT_{y}\left(p\right)$ is the distance transform of (DT) [26] of the binary edge image *y* and is defined as the distance from the pixel *p* to its closest edge point in *y*.

(2) $$DT_{y}(p)=\underset{q\in y}{\min}||p-q||$$

where ||.|| means the Euclidean distance. To cover a wide range of human postures, we construct a set of representative weighted contour templates $\Theta ={\left\{\lambda_{j},\theta_{j}\right\}}_{j=1}^{\left|\Theta \right|}$, where |Θ| denotes the number of representative weighted contour templates, $\lambda_{j}$ is the weight of the template $\theta_{j}$ and $\overset{\left|\Theta \right|}{\underset{j=1}{\sum }}\lambda_{j}$=1.

### 2.2. Expectation Maximization (EM)-Based Formulation

By formulating the template construction problem as the maximum likelihood one, an algorithm called (EM) [25] is adopted to obtain Θ without human intervention. Let $Y={\left\{y_{i},t_{i}\right\}}_{i=1}^{\left|Y\right|}$ be a set of |Y| training samples, where $y_{i}$ is the binary edge image of the *i*th sample and $t_{i}\in \left\{+1,-1\right\}$ is the ground-truth label of $y_{i}$. The binary edge images for training are all obtained by applying the Canny edge detector. Figure 1b are some training examples including both positive and negative ones. Based on the assumption that training samples are i.i.d (independent and identically distributed), the likelihood probability of Y given Θ can be defined as $\mathrm{Pr}(Y|\Theta )=\prod_{i=1}^{\left|Y\right|}\mathrm{Pr}(y_{i}|\Theta )$. Accordingly, the maximization of Pr(Y|Θ) leads to the construction of a set of weighted contour templates which is denoted as $\hat{\Theta }$. Since the sum operator is easier than product operator in implementation, it is often to calculate the $\hat{\Theta }$ that maximizes the log-likelihood of Y, that is,

(3) $$\hat{\Theta }=\arg\underset{\Theta }{\max}\log\mathrm{Pr}(Y|\Theta )=\arg\underset{\Theta }{\max}\sum_{i=1}^{\left|Y\right|}\log\mathrm{Pr}(y_{i}|\Theta )$$

Therefore, a latent random variable $Z={\left\{z_{i}\right\}}_{i=1}^{\left|Y\right|}$ is thus introduced to model the relation between Y and Θ, where $z_{i}\in \left\{1,2,...,|\Theta |\right\}$ is a discrete random variable that defines which template the observed image $y_{i}$ comes from. Given the observed data Y and currently estimated $\Theta^{\left(m\right)}$, we make a guess about Z and find the $\Theta^{\left(m+1\right)}$ that maximizes the log-expectation of Pr(Y,Z|Θ), which is called Q-function in the EM literature.

(4) $$Q(\Theta |\Theta^{\left(m\right)})=\int_{Z}\log(Y,Z|\Theta )\mathrm{Pr}(Z|Y,\Theta^{\left(m\right)})\;=\sum_{i=1}^{\left|Y\right|}\int_{z_{i}}\log\mathrm{Pr}(y_{i},z_{i}|\Theta )\mathrm{Pr}(z_{i}|y_{i},\Theta^{\left(m\right)})$$

The first term $\mathrm{Pr}(y_{i},z_{i}=j|\Theta )$ introduced in the right-hand site of (4) evaluates the possibility of the training sample is from the $z_{i}$th template. Since the template used is the human contour, the similarity evaluation between $y_{i}$ and $z_{i}$th template is utilized to define this term. Similar to the definition of Normal distribution, $\mathrm{Pr}(y_{i},z_{i}=j|\Theta )$ is defined as:

(5) $$\mathrm{Pr}(y_{i},z_{i}=j|\Theta )=\lambda_{j}\exp\left\{-\beta \times d(y_{i},\theta_{j})\right\}$$

where β is a parameter for controlling the effect of the matching distance and is set to 0.01. Remarkably, this term $\mathrm{Pr}(y_{i},z_{i}|\Theta )$ is a function of unknown parameter Θ. The second term $\mathrm{Pr}(z_{i}|y_{i},\Theta^{\left(m\right)})$ denotes the probability that the *i*th training image $y_{i}$ belongs to $z_{i}$th template based on the estimated $\Theta^{\left(m\right)}$ and can be evaluated as:

(6) $$\mathrm{Pr}(z_{i}=j|y_{i},\Theta^{\left(m\right)})=\frac{\lambda_{j}^{\left(m\right)}\exp\left\{-\beta \times d(y_{i},\theta_{j}^{\left(m\right)})\right\}}{\sum_{l=1}^{\left|\Theta \right|}\lambda_{l}^{\left(m\right)}\exp\left\{-\beta \times d(y_{i},\theta_{l}^{\left(m\right)})\right\}}$$

For notation simplicity, let $\gamma_{ij}^{\left(m\right)}=\mathrm{Pr}(z_{i}|y_{i},\Theta^{\left(m\right)})$ which satisfies $\sum_{j=1}^{\left|\Theta \right|}\gamma_{ij}^{\left(m\right)}=1$. The Q-function in (4) can be accordingly becomes:

(7) $$Q(\Theta |\Theta^{\left(m\right)})=\sum_{i=1}^{\left|Y\right|}\sum_{j=1}^{\left|\Theta \right|}\gamma_{ij}^{\left(m\right)}\left(-\beta \times d(y_{i},\theta_{j})+\log\lambda_{j}\right)$$

### 2.3. Template Construction Algorithm

After introducing the EM framework for formulating the problem of weighted template construction, we further elaborate the implementation issues in this section. Initially, an incremental clustering similar to [27] is firstly applied to generate a set of good initial templates. In this stage, all contour points in a template are set as equally important and have the same weights. After obtaining the initial templates, the E-Step and M-Step are performed iteratively until the convergence condition is reached. At each round *m*, the E-Step is to calculate the possibilities of all training samples derived from each weighted contour template at the current stage as the definition of $\gamma_{ij}^{\left(m\right)}$. According to the estimated $\gamma_{ij}^{\left(m\right)}$, the M-Step updates all weighted contour templates at the current stage denoted as $\Theta^{\left(m\right)}$ to obtain a set of new weighted contour templates $\Theta^{\left(m+1\right)}$ so that the Q-function is maximized. For each $\theta_{j}^{\left(m\right)}\in \Theta^{\left(m\right)}$, the associated template weight $\lambda_{j}$ is firstly updated as:

(8) $$\lambda_{j}^{\left(m\right)}=\frac{\sum_{i=1}^{\left|Y\right|}\gamma_{ij}^{\left(m\right)}}{\sum_{j=1}^{\left|\Theta \right|}\sum_{i=1}^{\left|Y\right|}\gamma_{ij}^{\left(m\right)}}$$

The update of template $\theta_{j}^{\left(m\right)}$ to $\theta_{j}^{\left(m+1\right)}$ starts from the determination of the number of contour points in $\theta_{j}^{\left(m+1\right)}$. It is defined as the weighted sum of point numbers in all positive samples with respect to $\theta_{j}^{\left(m\right)}$.

(9) $$\left|\theta_{j}^{\left(m+1\right)}\right|=\frac{\sum_{y_{i}\in Y^{+}}\gamma_{ij}^{\left(m\right)}\times \left|y_{i}\right|}{\sum_{y_{i}\in Y^{+}}\gamma_{ij}^{\left(m\right)}}$$

where $\left|y_{i}\right|$ is the number of contour points in the training image $y_{i}$. The next step is to localize all contour points. Let $c_{j}(m,n)$ denote the confidence value of the point (m,n) belonging to the contour point. Here, $c_{j}(m,n)$ is defined as:

(10) $$c_{j}(m,n)=\sum_{y_{i}\in Y^{+}}\gamma_{ij}^{\left(m\right)}\times b_{i}(m,n)$$

where $Y^{+}=\left\{y_{i}|t_{i}=+1\right\}$ is the set of the positive training images and $b_{i}(m,n)$ denotes whether the point (m,n) of the training image $y_{i}$ is a contour point $b_{i}(m,n)=1$ or not $b_{i}(m,n)=0$. By sorting the points in descending order according to their confidence values, we label the first $\left|\theta_{j}^{\left(m+1\right)}\right|$ points labelled as the contour ones.

**Figure 2:**
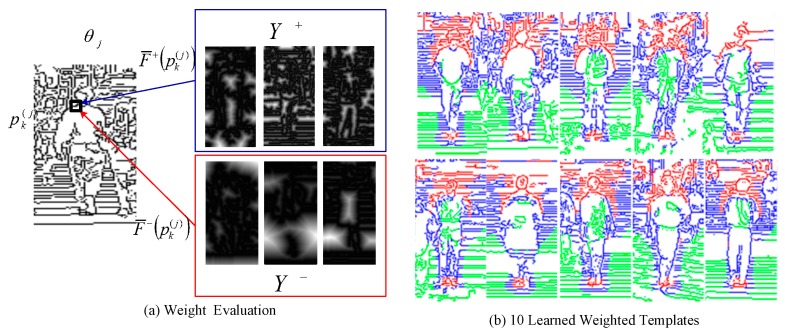
(**a**) Schematic description for weight evaluation; (**b**) is the learned 10 templates using the expectation maximization (EM) algorithm. Red, blue, and green points are with high-weight, middle-weight and low-weight, respectively.

The last step is to determine the weight (importance) $\alpha_{k}^{\left(j\right)}$ depending on its power in distinguishing human from non-human. Let ${\bar{F}}^{+}(p_{k}^{\left(j\right)})$ and ${\bar{F}}^{-}(p_{k}^{\left(j\right)})$ be the average matching distances of the positive and negative training sets, $Y^{+}$ and $Y^{-}$, respectively, to the contour point $p_{k}^{\left(j\right)}$. The formal definition of ${\bar{F}}^{+}(p_{k}^{\left(j\right)})$ and ${\bar{F}}^{-}(p_{k}^{\left(j\right)})$ is given in (11) and the illustration of weight evaluation in the schematic form is shown in Figure 2a.

(11) $${\bar{F}}^{+}(p_{k}^{\left(j\right)})=\frac{1}{\left|Y^{+}\right|}\sum_{y_{i}\in Y^{+}}\gamma_{ij}^{\left(m\right)}DT_{y_{i}}(p_{k}^{\left(j\right)})\;{\bar{F}}^{-}(p_{k}^{\left(j\right)})=\frac{1}{\left|Y^{-}\right|}\sum_{y_{i}\in Y^{-}}\gamma_{ij}^{\left(m\right)}DT_{y_{i}}(p_{k}^{\left(j\right)})$$

Here, the contrast value of ${\bar{F}}^{+}(p_{k}^{\left(j\right)})$ and ${\bar{F}}^{-}(p_{k}^{\left(j\right)})$ is utilized to define the weight as:

(12) $$\alpha_{k}^{\left(j\right)}=\frac{1}{1+\exp\left\{\frac{{\bar{F}}^{+}(p_{k}^{\left(j\right)})-{\bar{F}}^{-}(p_{k}^{\left(j\right)})}{{\bar{F}}^{+}(p_{k}^{\left(j\right)})+{\bar{F}}^{-}(p_{k}^{\left(j\right)})}\right\}}$$

The larger $\alpha_{k}^{\left(j\right)}$ is, the more important the point $p_{k}^{\left(j\right)}$ is in human/non-human discrimination. When ${\bar{F}}^{+}(p_{k}^{\left(j\right)})>={\bar{F}}^{-}(p_{k}^{\left(j\right)})$, $\alpha_{k}^{\left(j\right)}$ is less than or equals 0.5 and the point $p_{k}^{\left(j\right)}$ has no matching contribution. Algorithm 1 gives the pseudo code of detailed implementation.

**Algorithm 1:** Algorithm for Weighted Template Construction
**Input:** A set of training samples $Y={\left\{y_{i},t_{i}\right\}}_{i=1}^{\left|Y\right|}$
**Output:** A set of weighted templates $\Theta ={\left\{\lambda_{j},\theta_{j}\right\}}_{j=1}^{\left|\Theta \right|}$
Apply the distance transform to all training samples.Take the samples in positive set *Y*^+^ to generate a set of |Θ| initial templates ${\left\{\theta_{j}\right\}}_{j=1}^{\left|\Theta \right|}$ by using incremental clusteringSet all template weights to $\lambda_{j}=\frac{1}{\left|\Theta \right|}\;\mathrm{and}\;m\leftarrow 0$

**repeat**
 **E-Step:** Form log expected function
Calculate $\gamma_{ij}^{\left(m\right)}$ for 1≤i≤|Y| and 1≤j≤|Θ|Form the Q-function defined in (7) **M-Step:** Update the parameters as follows to maximize the Q-function.**for**j=1 to |Θ|(1)Update $\lambda_{j}^{\left(m+1\right)}$ according to (8)(2)Determine the number of contour points $\theta_{j}^{\left(m+1\right)}$ and their positions $\left\{p_{k}^{\left(j\right)}\right\}$ according to (9) and (10), respectively.(3)Assign a weight $\left\{\alpha_{k}^{\left(j\right)}\right\}$ to each contour point according to (12)**end for**

 *m* ← *m*+1

**Until** $\left|\left|\Theta^{\left(m+1\right)}-\Theta^{\left(m\right)}\right|\right|<\delta$



### 2.4. Classifier Formation and Analysis

**Figure 3:**
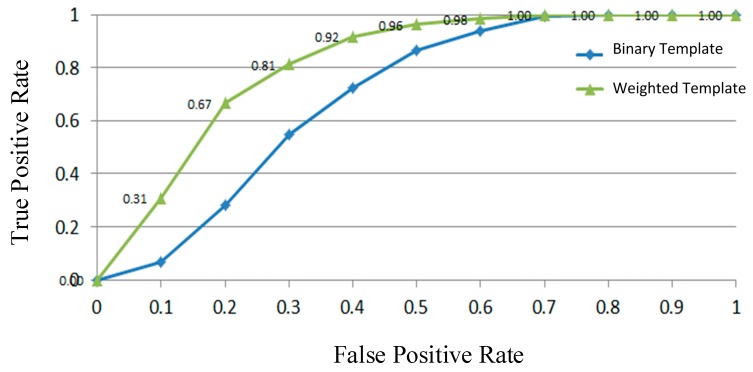
Performance comparison of the proposed global classifier $H_{G}\left(.\right)$ (green curve) with that (blue curve) only using binary contour templates for human classification.

In this section, we describe how to learn a classifier based on a set of weighted contour templates and analyze the performance improvement in imposing the weight to every contour point. The dataset used consists of 924 positive subjects from the MIT CBCL dataset [28] and 3342 negative ones from the INIRA dataset [29]. First of all, a half of dataset is considered as training dataset and is used to construct a set of weighted contour templates. The generated 10 weighted contour templates through EM algorithm are shown in Figure 2b. The high-weight contour points are labelled in red color and obviously locate at the salient body part, such as head or shoulder. The low-weight contour points with green color are at the background edges or in the interior of body part. This exhibits that the weighted contour templates constructed by the proposed EM algorithm are effective in representing the contour of a human. A classifier $H_{G}(.)$ called global classifier based on the constructed weighted contour templates $\hat{\Theta }$ to determine the existence of the human is thus defined as:

(13) $$H_{G}(y)=\{\begin{array}{ccc} \begin{array}{c} (+1)\;\mathrm{Human}\\ (-1)\mathrm{Non}-\mathrm{Human} \end{array} & if & \begin{array}{c} \underset{}{\underset{\theta_{j}\in \Theta }{\min}d(y,\theta_{j})<Th_{G}}\\ \mathrm{otherwise} \end{array} \end{array}$$

where $TH_{G}$ is a threshold and is set as the value that minimizes the training error. The learned classifier $H_{G}\left(.\right)$ is thus applied to another half part of dataset, called testing dataset, for analysis. For validating the effectiveness of imposing the weight to every contour point, $H_{G}\left(.\right)$ is compared with the approach only using binary templates which considers the contour points as equally weighted. Figure 3 exhibits the ROC (receiver operating characteristic) curves of proposed classifier $H_{G}\left(.\right)$ using weighted contour templates and the one using binary templates. Obviously, the proposed classifier $H_{G}\left(.\right)$ has superior performance.

## 3. Training Framework

HOGs proposed by [14] are an effective feature to represent the human appearance in a local patch. The description of the human appearance is simply achieved by the concatenation of thousands of local HOGs and a SVM classifier is trained for human and non-human discrimination in such high-dimensional feature space. To reduce time complexity of detection process, the work in [30] learns a SVM classifier for each patch representing by HOGs feature and uses boosting algorithm to select a set of SVM classifiers to form a human detector. Boosting is a way to approach the solution by iteratively reducing training error with a set of additive classifiers. However, HOGs as a kind of local feature generally suffer from the false detection problem in case of complex background or noise effect. Motivated by [31], the way to alleviate this problem is by imposing the learned classifier $H_{G}(.)$ to Zhu’s [30] boosting framework. This integrates the global contour and local HOGs features so that the detection accuracy can be improved.

### 3.1. Biased Boosting

First of all, we briefly describe Zhu’s boosting framework for the learning of a human detector in this section. Let H be a set of learned SVM classifiers in each of which h∈H is referred to as weak classifier in the boosting literature. Initially, each training sample $y_{i}$ is assigned a weight $D_{i}^{\left(0\right)}$ which represents a level of classification difficulty. Let $\xi^{\left(m\right)}$ be the error rate of $h^{\left(m\right)}$ at the round m over all training samples and is defined as:

(14) $$\xi^{\left(m\right)}=\sum_{i=1}^{\left|Y\right|}D_{i}^{\left(m\right)}1_{t_{i}\neq h^{\left(m\right)}(y_{i})}$$

where 1. is an indicator function. The selected weak classifier ${\tilde{h}}^{\left(m\right)}$ is the one which has minimal training error. The form of ${\tilde{h}}^{\left(m\right)}$ for human and non-human discrimination using SVM can be formally expressed as:

(15) $${\tilde{h}}^{\left(m\right)}=\{\begin{array}{ccc} \begin{array}{c} \left(+1\right)\\ \left(-1\right) \end{array} & if & \begin{array}{c} \underset{}{\phi_{svm}^{\left(m\right)}(y)\geq 0}\\ \underset{}{\phi_{svm}^{\left(m\right)}(y)<0} \end{array} \end{array}$$

where $\phi_{svm}^{\left(m\right)}(.)$ is a SVM hyper-plane which makes decision based on a specific local HOGs patch of $y_{i}$. The confidence $\pi^{\left(m\right)}$ of the selected weak classifier ${\tilde{h}}^{\left(m\right)}$ is set as:

(16) $$\pi^{\left(m\right)}=\frac{1}{2}\ln\left(\frac{1-\xi^{\left(m\right)}}{\xi^{\left(m\right)}}\right)$$

The weight $D_{i}^{\left(m\right)}$ of each training sample $y_{i}$ is updated accordingly as:

(17) $$D_{i}^{\left(m+1\right)}=D_{i}^{\left(m\right)}\exp\left(-\pi^{\left(m\right)}\times t_{i}{\tilde{h}}^{\left(m\right)}(y_{i})\right)$$

The integration of the global contour with local HOGs features is thus by adjusting the bias of the SVM classifier at each round m. For the samples classified as human ones by $H_{G}(.)$, they are generally with a human-like contour and have high possibility of the ground-true labels equal to positive (human). To response this, we move the $\phi_{svm}^{\left(m\right)}(.)$ hyper-plane $Th_{G^{+}}^{\left(m\right)}$ towards negative margin and result in a positive-biased weak classifier ${\tilde{h}}^{+(m)}$ for the samples $G^{+}=\{y_{i}|H_{G}(y_{i})=+1\}$. This corrects the mis-classified ones to positive so as to improve the detection rate. By contrast, to remove the false detections resulting from complex background and noise effect, we move $\phi_{svm}^{\left(m\right)}(.)$ hyper-plane $Th_{G^{-}}^{\left(m\right)}$ towards positive margin for the samples $G^{-}=\{y_{i}|H_{G}(y_{i})=-1\}$ to obtain a negative-based classifier ${\tilde{h}}^{-(m)}$. Accordingly, the formal definitions of ${\tilde{h}}^{+(m)}$ and ${\tilde{h}}^{-(m)}$ can be expressed as:

(18) $${\tilde{h}}^{+(m)}(y)=\{\begin{array}{ccc} \begin{array}{c} \left(+1\right)\\ \left(-1\right) \end{array} & if & \begin{array}{c} \phi_{svm}^{\left(m\right)}(y)\geq -Th_{G^{+}}^{\left(m\right)}\\ \mathrm{otherwise} \end{array} \end{array}\;{\tilde{h}}^{-(m)}(y)=\{\begin{array}{ccc} \begin{array}{c} \left(+1\right)\\ \left(-1\right) \end{array} & if & \begin{array}{c} \phi_{svm}^{\left(m\right)}(y)\leq +Th_{G^{-}}^{\left(m\right)}\\ \mathrm{otherwise} \end{array} \end{array}$$

In short, the weak classifier ${\tilde{h}}^{\left(m\right)}$ in the original boosting framework is decomposed to ${\tilde{h}}^{+(m)}$ and ${\tilde{h}}^{-(m)}$, respectively, $G^{+}$ and $G^{-}$, as:

(19) $${\tilde{h}}^{\left(m\right)}(y)=\{\begin{array}{ccc} \begin{array}{c} {\tilde{h}}^{+(m)}(y)\\ {\tilde{h}}^{-(m)}(y) \end{array} & if & \begin{array}{c} H_{G}(y)\in +1\\ H_{G}(y)\in -1 \end{array} \end{array}$$

Thus, the mis-classification $\xi_{bias}^{\left(m\right)}$ over all training samples is re-expressed as:

(20) $$\xi_{bias}^{\left(m\right)}=\sum_{y_{i}\in G^{+}}D_{i}^{\left(m\right)}\times 1_{t_{i}\neq {\tilde{h}}^{+(m)}(y_{i})}+\sum_{y_{i}\in G^{-}}D_{i}^{\left(m\right)}\times 1_{t_{i}\neq {\tilde{h}}^{-(m)}(y_{i})}$$

And each sample weight is updated as:

(21) $$D_{bias,i.}^{\left(m+1\right)}=\{\begin{array}{ccc} \begin{array}{c} D_{bias,i}^{\left(m\right)}\exp\left(-\pi^{\left(m\right)}\times t_{i}{\tilde{h}}^{+(m)}(y_{i})\right)\\ D_{bias,i}^{\left(m\right)}\exp\left(-\pi^{\left(m\right)}\times t_{i}{\tilde{h}}^{-(m)}(y_{i})\right) \end{array} & if & \begin{array}{c} y_{i}\in G^{+}\\ y_{i}\in G^{-} \end{array} \end{array}$$

Finally, we obtain the human detector consisting of $H_{G}(.)$ and ${\left\{\pi^{\left(m\right)},{\tilde{h}}^{\left(m\right)},Th_{G^{+}}^{\left(m\right)},Th_{G^{-}}^{\left(m\right)}\right\}}_{m=1}^{M}$. The pseudo code of the proposed biased boosting is given in Algorithm 2. Obviously, the two bias values $Th_{G^{+}}^{\left(m\right)}$ and $Th_{G^{-}}^{\left(m\right)}$ will significantly affect the detection performance of ${\tilde{h}}^{\left(m\right)}$ and their determination will be deferred to the next section.

**Algorithm 2:** Biased Boosting Algorithm
**Input:** A set of training samples $Y={\left\{y_{i},t_{i}\right\}}_{i=1}^{\left|Y\right|}$

**Output:** ${\left\{\pi^{\left(m\right)},{\tilde{h}}^{\left(m\right)},Th_{G^{+}}^{\left(m\right)},Th_{G^{-}}^{\left(m\right)}\right\}}_{m=1}^{M}$
Initialize the positive sample weight to $\frac{1}{\left|Y^{+}\right|}$ and negative sample weight to $\frac{1}{\left|Y^{-}\right|}$**for***j* = 1 to M
(1)Find the classifier ${\tilde{h}}^{\left(m\right)}$ that has the minimal error defined in (14)(2)Estimate two bias values $Th_{G^{+}}^{\left(m\right)}\;\mathrm{and}\;Th_{G^{-}}^{\left(m\right)}$ using bias determination strategy discussed in Section 3.2.(3)Calculate the error rate $\xi^{\left(m\right)}$ in (20) over all samples and estimate the importance $\pi^{\left(m\right)}$ in (16).(4)Update the sample weight $D_{i}^{\left(m\right)}$ according to (21)
(1)Update $\lambda_{j}^{\left(m+1\right)}$ according to (8)(2)Determine the number of contour points $\theta_{j}^{\left(m+1\right)}$ and their positions $\left\{p_{k}^{\left(j\right)}\right\}$ according to (9) and (10), respectively.(3)Assign a weight $\left\{\alpha_{k}^{\left(j\right)}\right\}$ to each contour point according to (12)

 **end for**


### 3.2. Bias Determination

**Figure 4:**
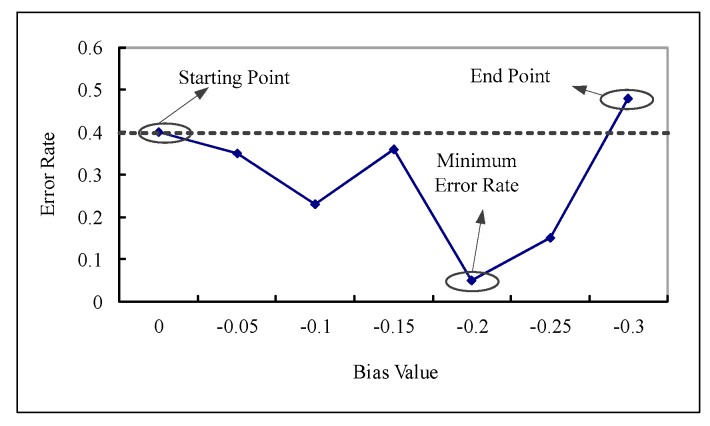
Bias determination: the horizontal axis denotes the bias increment; the vertical one denotes the resulting error rate.

The main concept of boosting is to choose a weak classifier at each round *m* so as to maximally reduce the error rate on the weighted training set. To conform to this, a strategy for searching appropriate bias values is proposed and described as follows. We adjust $Th_{G^{+}}^{}$ and $Th_{G^{-}}^{}$ to lower down the total error rate $\xi^{}$ by increasing with interval 0.05. If the error of the biased weak classifier exceeds in the initial error (obtained from the basis of $Th_{G^{+}}^{}$ = $Th_{G^{-}}^{}$ = 0.0), the searching process should be stopped. Thus, the value that derives the lowest error rate within the searching interval is taken as the final bias. Figure 4 illustrates the proposed strategy for bias determination.

### 3.3. Bias Determination

**Figure 5:**
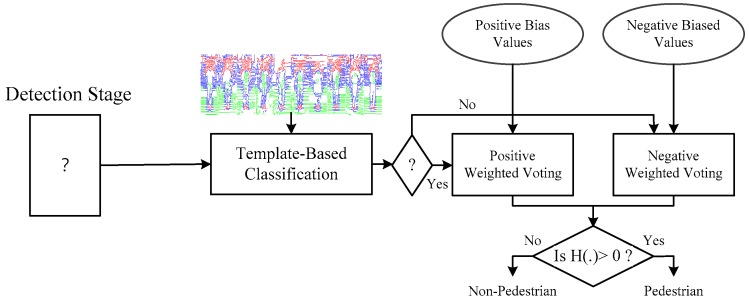
Detection stage: the process to detect the human using the learned detector from biased boosting.

In this section, we describe how to determine the existence of the human of a scanning window y in an image using the learned detector H(.). The first step is to check if the appearance of y has a human-like contour. If the answer is yes, the set of positive-based weak classifiers ${\left\{\pi^{\left(m\right)},{\tilde{h}}^{\left(m\right)},Th_{G^{+}}^{\left(m\right)}\right\}}_{m=1}^{M}$ is used for further classification; otherwise, the set of negative-based weak classifiers ${\left\{\pi^{\left(m\right)},{\tilde{h}}^{\left(m\right)},Th_{G^{-}}^{\left(m\right)}\right\}}_{m=1}^{M}$ is used. The flow chart of the detection process is illustrated in Figure 5. The formal definition of the final human detector can be expressed as:

(22) $$D_{i}^{\left(m+1\right)}=\{\begin{array}{ccc} \begin{array}{c} sign\left(\sum_{m=1}^{M}\pi^{\left(m\right)}{\tilde{h}}^{+(m)}(y)\right)\\ sign\left(\sum_{m=1}^{M}\pi^{\left(m\right)}{\tilde{h}}^{-(m)}(y)\right) \end{array} & if & \begin{array}{c} H_{G}(y)=+1\\ H_{G}(y)=-1 \end{array} \end{array}$$

## 4. Experiment

To validate the effectiveness of the proposed method called WTM-Boost, we implement three methods proposed in [14] (HOG-SVM), [3] (WTM), and [32] (TM-Boost) for comparison. We consider these three algorithms for comparison because HOG-SVM uses HOG local features, WTM is based on weighted templates which is a kind of global feature, TM-Boost combines local and global features but only use binary contour templates instead of weighted contour ones. The templates in our work and [32] are both learned from the EM algorithm but those used in the work [32] are binary contour templates all the points of which are considered as equally weighted in matching. The templates used in [3] are formed by using k-means clustering algorithm with k = 10 in order to obtain the same number of templates used in our work and [32]. In [14], the human appearance is described by the dense HOGs and a SVM classifier is learned for human detection. In implementation, the parameter settings in HOGs representation and SVM classifier learning for these three methods are all the same in this work. The sizes of HOGs blocks used are 16, 24, 36, 48, and 60, and the aspect ratio for each block can be one of the following choices: (1:1), (1:2), and (2:1).

**Figure 6:**
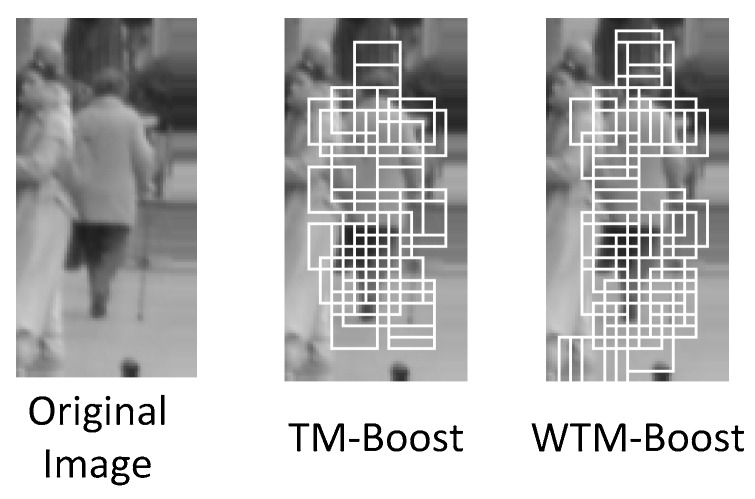
The 40 learned weak classifiers for TM-Boost and WTM-Boost methods, respectively.

The cost constant for training the SVM as the weak classifier for a block is 1.0 and the kernel function is Gaussian radius basis one. The number of weak classifiers used in boosting framework is 40 in all methods. The white rectangles in Figure 6 shows the learned 40 weak classifiers from biased boosting for TM-Boost and WTM-Boost, respectively. The aforementioned methods are implemented in C programming language with the support of OpenCV library and are then run on a computer with Intel i7 3.4GHz and 8GB RAM. In this work, no GPU is used for speeding up. Table 1 lists the average processing time of all testing samples for the four implemented methods at different stages, respectively. Since HOG-SVM describes human appearance in dense manner, it wastes more time in HOGs computation than TM-Boost and WTM-Boost. However, HOG-SVM performs SVM classification more efficiently than TM-Boost and WTM-Boost which have to 40 weak classifiers.

**Figure 7:**
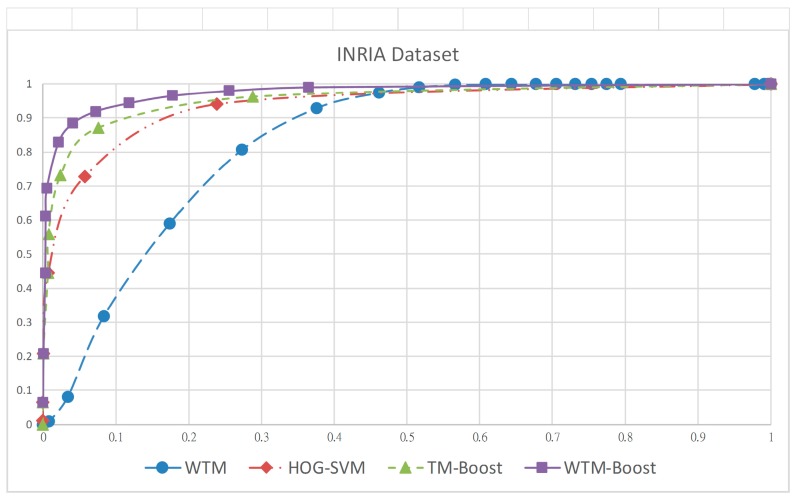
Receiver operating characteristics (ROC) curve for four approaches, WTM, HOG-SVM, TM-Boost, and WTM-Boost on INRIA dataset.

**Figure 8:**
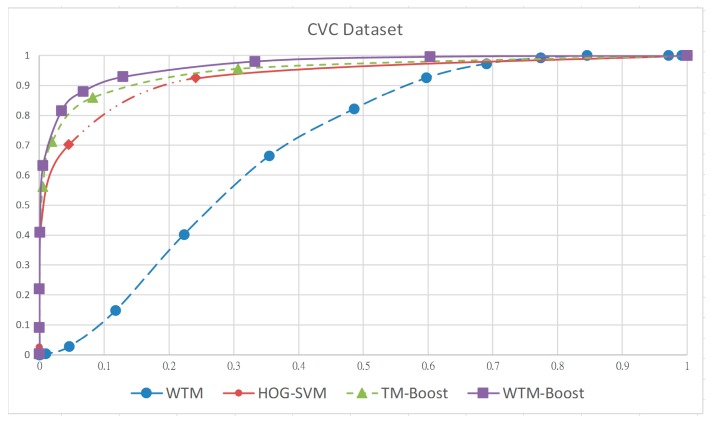
ROC curve for four approaches, WTM, HOG-SVM, TM-Boost, and WTM-Boost on CVC dataset.

For performance validation, we use three popular human datasets including MIT CBCL, INRIA, and CVC in our experiment. The statistics of images from the three datasets for training and testing is listed in Table 2. Of all the training samples, all 924 human images in the CBCL dataset are provided as positive samples, while the negative samples come from 3342 randomly-chosen images from the INRIA dataset because there are no non-human images in CBCL dataset. The training dataset is used for weighted template construction as well as detector boosting. For validating the trained detector in experiments I and II at the testing stage, the positive and negative images are, respectively, from the INRIA and CVC datasets. The ROC (receiver operating characteristics) curve which illustrates the relation of detection rate and false positive rate is used for objective evaluation. The four curves, respectively, shown in Figure 7 and Figure 8 are those of the ROC for the INRIA and CVC datasets of the four methods. Obviously, detectors learned by machine learning algorithm, such as boosting and SVM, have superiority over the template-matching algorithm in both datasets. This is because a few of used templates is hard to model the significant appearance variation in human pose. The curves of the proposed WTM-Boost method for both datasets are closer to the top-left hand and exhibit better performance. Imposing the contour template to the boosting framework makes the global contour and local HOGs features complement each other in a mutually beneficial manner so that TM-Boost as well as WTM-Boost methods outperforms the HOG-SVM one. Besides, using the weighted contour template to describe the human appearance in various poses is more effective than the binary one and this is the reason why the proposed WTM-Boost has better accuracy than TM-Boost.

To further validate this point, we replace the training samples from the MIT CBCL dataset with those from the INRIA and CVC datasets, respectively, for experiments I and II, to construct the templates for matching in the TM-Boost method. The resulting templates are for global classifier learning followed by boosting the human detector, as mentioned. The ROC curves of the human detector learned using TM-Boost for INRIA and CVC datasets are shown in Figure 7 and Figure 8, respectively. Obviously, the accuracy is almost the same as to WTM-Boost. This indicates that the performance difference between TM-Boost and WTM-Boost is from the representation ability of their used templates. In other word, the proposed WTM-boost method can alleviate the overfitting problem because it uses the weight(s) assigning to the contour point(s).

## 5. Conclusions

The main contribution of our work lies in two aspects. Firstly, we propose a method based on the EM algorithm to automatically construct a set of representatively weighted contour templates By formulating the problem of template construction as a maximum likelihood one, the contour template as well as contour point weight are determined in the M-Step according to the estimated likelihood probabilities of all training samples in the E-Step. The assignment of different weights to the contour points gives the constructed templates more discriminative power.

Secondly, we systematically integrate the global contour and local HOGs features in the proposed biased boosting framework. The determination of bias values, respectively, for those with contours similar to and different from the pedestrian templates, is by finding the values minimizing the error rate. By comparing the other two approaches, the experimental results exhibit that the trained pedestrian detector increases the detection rate and reduces the false positive rate as well. Given the effectiveness and power of deep learning, the use of deep learning is the trend in the detection area [33,34]. One of the main advantages of deep learning is to extract the semantic features through the convolution and pooling layers. Since our proposed boosting framework is to integrate various features, it could be used to fuse the extracted feature from deep learning in our future work.

## Figures and Tables

**Table 1:** Processing time analysis for a testing sample (64 × 128).

(ms)	WTM	HOG-SVM	TM-Boost	WTM-Boost
Distance Transform Matching Process	15.6 15.3	x x	15.6 14.5	15.6 15.4
HOG Descriptor SVM Classification	x x	24.1 0.5	11.1 2.9	10.9 3.1
Total Time	30.9	24.6	44.1	45.0

**Table 2:** Image statistics for training and testing.

	Training		Testing	
	Positive	Negative	Positive	Negative
Exp I	CBCL (924)	INRIA (3342)	INRIA (2416)	INRIA (5561)
Exp II	CBCL (924)	INRIA (3342)	CVC (3356)	INRIA (8823)

## References

[1] Enzweiler M., Gavrila D.M. (2009). Monocular Pedestrian Detection: Survey and Experiments. IEEE Trans. Pattern Recognit. Mach. Intell..

[2] Gavrila D.M. (2007). A Bayesian, Exemplar-Based Approach to Hierarchical Shape Matching. IEEE Trans. Pattern Recognit. Mach. Intell..

[3] Thanh N.D., Li W., Ogunbona P. A Novel Template Matching Method for Human Detection. Proceedings of the 2009 16th IEEE International Conference on Image Processing (ICIP).

[4] Wang G., Liu Q., Zheng Y., Peng S. Far-Infrared Pedestrian Detection Based on Adaptive Template Matching and Heterogeneous-Feature-Based Classification. Proceedings of the 2016 IEEE International Instrumentation and Measurement Technology Conference Proceedings.

[5] Arie M., Shibata M., Terabayashi K., Moro A. Fast Human Detection Using Template Matching for Gradient Images and ASC Descriptors Based on Subtraction Stereo. Proceedings of the 2013 IEEE International Conference on Image Processing.

[6] Wu P., Cao X.-B., Xu Y.-W., Qiao H. Representative Template Set Generation Method for Pedestrian Detection. Proceedings of the 2008 Fifth International Conference on Fuzzy Systems and Knowledge Discovery.

[7] Rogez G., Rihan J., Orrite-Urunuela C., Torr P.H.S. (2012). Fast Human Pose Detection Using Randomized Hierarchical Cascades of Rejectors. Int. J. Comput. Vis..

[8] Hao Z., Wang B., Teng J. (2010). Fast Pedestrian Detection Based on Adaboost and Probability Template Matching. IEEE Int. Adv. Comput. Control.

[9] Nguyen T., Ogunbona D.P., Li W. Human Detection Based on Weighted Template Matching. Proceedings of the 2009 IEEE International Conference on Multimedia and Expo.

[10] Lee H.J., Hong K.-S. (2012). Class-Specific Weighted Dominant Orientation Templates for Object Detection. Asian Conference on Computer Vision.

[11] Hinterstoisser S., Lepetit V., Ilic S., Fua P., Navab N. Dominant Orientation Templates for Real-Time Detection of Texture-Less Objects. Proceedings of the 2010 IEEE Computer Society Conference on Computer Vision and Pattern Recognition.

[12] Han H., Fan Y., Jiao L., Chen Z. Concatenated Edge and Co-occurrence Feature Extracted from Curvelet Transform for Human Detection. Proceedings of the 2010 25th International Conference of Image and Vision Computing New Zealand.

[13] Zeng C., Ma H. Robust Head-Shoulder Detection by PCA-Based Multi-Level HOG-LBP Detector for People Counting. Proceedings of the 2010 20th International Conference on Pattern Recognition.

[14] Dalal N., Triggs B. Histograms of Oriented Gradients for Human Detection. Proceedings of the International Conference on Computer Vision & Pattern Recognition.

[15] Paisitkriangkrai S., Shen C., Zhang J. (2008). Performance Evaluation of Local Features in Human Classification and Detection. IET Comput. Vis..

[16] Wang C.C.R., Lien J.J. (2007). AdaBoost Learning for Human Detection Based on Histograms of Oriented Gradients. Asian Conference on Computer Vision.

[17] Chuang C.H., Huang S.S., Fu L.C., Hsiao P.Y. Monocular Multi-Human Detection Using Augmented Histograms of Oriented Gradients. Proceedings of the 2008 19th International Conference on Pattern Recognition.

[18] Ojala T., Pietikäinen M., Mäenpää T. (2002). Multi-Resolution Gray-Scale and Rotation Invariant Texture Classification with Local Binary Pattern. IEEE Trans. Pattern Anal. Mach. Intell..

[19] Wu B., Nevatia R. Detectiong of Multiple, Partially Occluded Humans in a Single Image by Bayesian Combination of Edgelet Part Detectors. Proceedings of the Tenth IEEE International Conference on Computer Vision (ICCV’05).

[20] Wu B., Nevatia R. Simultaneous Object Detection and Segmentation by Boosting Local Shape Feature Based Classifier. Proceedings of the 2007 IEEE Conference on Computer Vision and Pattern Recognition.

[21] Sabzmeydani P., Mori G. Detecting Pedestrians by Learning Shapelet Features. Proceedings of the 2007 IEEE Conference on Computer Vision and Pattern Recognition.

[22] Hurney P., Waldron P., Morgan F., Jones E., Glavin M. (2015). Night-Time Pedestrian Classification with Histograms of Oriented Gradients-Local Binary Patterns Vectors. IET Trans. Intell. Transp. Syst..

[23] Yao S., Pan S., Wang T., Zheng C., Shen W., Chong Y. (2015). A New Pedestrian Detection Method Based on Combined HOG and LSS Features. Elsevier Neurocomput..

[24] Wang X., Han T.X., Yan S. An HOG-LBP Human Detection with Partial Occlusion Handling. Proceedings of the 2009 IEEE 12th International Conference on Computer Vision.

[25] Bilmes J.A. (1998). A Gentle Tutorial of the EM Algorithm and its Application to Parameter Estimation for Gaussian Mixture and Hidden Markov Models. Int. Comput. Sci. Inst..

[26] Borgefors G. (1986). Distance Transform in Digital Images. Comput. Vis. Graph. Image Process..

[27] Young S., Arel I., Karnowski T.P., Rose D. A Fast and S Incremental Clustering Algorithm. Proceedings of the IEEE International Conference on Information Technology.

[28] Pedestrian Data. http://cbcl.mit.edu/software-datasets/PedestrianData.html.

[29] INRIA Person Dataset. http://pascal.inrialpes.fr/data/human/.

[30] Zhu Q., Avidan S., Yeh M.C., Cheng K.T. Fast Human Detection Using a Cascade of Histograms of Oriented Gradients. Proceedings of the IEEE International Conference on Computer Vision and Pattern Recognition.

[31] Khoussainov R., He A., Kushmerick N. Ensembles of Biased Classifiers. Proceedings of the 22nd International Conference on Machine Learning.

[32] Huang S.S., Mao C.Y., Hsiao P.Y. Global Template Matching for Guiding the Learning of Human Detector. Proceedings of the 2012 IEEE International Conference on Systems, Man, and Cybernetics (SMC).

[33] Ren S., He K., Girshick R., Sun J. (2015). Faster R-CNN: Towards real-time object detection with region proposal networks. Advances in Neural Information Processing Systems 28, Proceedings of the Neural Information Processing Systems 2015, Montréal, QC, Canada, 7–12 December 2015.

[34] Redmon J., Farhadi A. YOLO9000: Better, faster, stronger. Proceedings of the IEEE Conference on Computer Vision and Pattern Recognition.

